# Circulating Levels of MicroRNAs Associated With Hypertension: A Cross-Sectional Study in Male and Female South African Participants

**DOI:** 10.3389/fgene.2021.710438

**Published:** 2021-09-14

**Authors:** Don M. Matshazi, Cecil J. Weale, Rajiv T. Erasmus, Andre P. Kengne, Saarah F. G. Davids, Shanel Raghubeer, Glenda M. Davison, Tandi E. Matsha

**Affiliations:** ^1^SAMRC/CPUT/Cardiometabolic Health Research Unit, Department of Biomedical Sciences, Faculty of Health and Wellness Science, Cape Peninsula University of Technology, Cape Town, South Africa; ^2^Division of Chemical Pathology, Faculty of Health Sciences, National Health Laboratory Service (NHLS) and Stellenbosch University, Cape Town, South Africa; ^3^Non-Communicable Diseases Research Unit, South African Medical Research Council, Cape Town, South Africa; ^4^Department of Medicine, University of Cape Town, Cape Town, South Africa

**Keywords:** hypertension, microRNA, RT-qPCR, Africa, blood pressure, non-coding RNA

## Abstract

MicroRNAs are non-coding, post-transcriptional regulators of gene expression and their dysregulation has been associated with development of various diseases, including hypertension. Consequently, understanding their role in the pathogenesis and progression of disease is essential. Prior research focusing on microRNAs in disease has provided a basis for understanding disease prognosis and offered possible channels for therapeutic interventions. Herein, we aimed to investigate possible differences in the expression profiles of five microRNAs in the blood of participants grouped on the basis of their hypertension status. This was done to elucidate the possible roles played by these microRNAs in the development of hypertension. Using quantitative reverse transcription polymerase chain reaction, we evaluated the expression levels of miR-126-3p, 30a-5p, 182-5p, 30e-3p, and 1299 in the whole blood of 1456 participants, normotensive (*n* = 573), screen-detected hypertensive (*n* = 304) and known hypertensive (*n* = 579). The expression of miR-126-3p and 182-5p was significantly higher in known hypertensives relative to both screen-detected hypertensives and normotensives, and also in screen-detected hypertensives vs normotensives. A significant association between the expression of miR-126-3p, 182-5p, and 30a-5p and known hypertension was also evident. This study demonstrated dysregulated miR-126-3p, 182-5p, and 30a-5p expression in hypertension, highlighting the possible efficacy of these microRNAs as targets for the diagnosis of hypertension as well as the development of microRNA-based therapies.

## Introduction

Hypertension (HPT) is a complex and multifactorial disease responsible for considerable loss of life globally (Campbell et al., 2016a; Mills et al., 2020). It is an important, modifiable risk factor for cardiovascular disease [1, 3], whose prevalence varies globally, but is on an upward trajectory in sub-Saharan Africa (Campbell et al., 2016a; Mills et al., 2016). The prevalence rates of HPT in some sub-Saharan African countries currently rank among the highest on the globe, in stark contrast to the picture from a few decades ago when the region had the lowest blood pressure levels (Danaei et al., 2011). Inroads into understanding the pathophysiology of HPT and advancing treatment options have been made over the years. The pathogenesis has been linked to various biological processes, including endothelial dysfunction, impaired angiogenesis, dysregulation of the renin-angiotensin-aldosterone axis and platelet activation (Taddei et al., 2001; Gkaliagkousi et al., 2010; Touyz et al., 2018). Sizeable financial investments have been made to study the genetic and environmental determinants of HPT and some of these studies have linked the Liddle phenotype (a hereditary disorder characterized by overactivity of the renal tubular epithelial sodium channel as a result of mutations in the *SCNN1B* and *SCNN1G* genes), and salt and water retention to the high HPT prevalence levels currently observed in sub-Saharan Africa (Spence and Rayner, 2018). However, in up to 95% of HPT cases, the etiology remains unknown and primary HPT continues to be a leading cause of morbidity and premature mortality globally (Carretero and Oparil, 2000; He and Macgregor, 2007). Described as a “silent killer,” its symptoms manifest in the later stages of the condition when hypertension-mediated target organ damage has possibly taken place (Moore, 2005).

Processes involved in blood pressure homeostasis are tightly regulated by various systems in the body. Amongst others, cellular processes like differentiation, growth and metabolism, are known to be under the control of microRNAs (miRNAs) (Vidigal and Ventura, 2015). These miRNAs are 18-25 nucleotide long, non-coding, post-transcriptional regulators of gene expression and their dysregulation has been linked to the development of cancer, essential HPT, viral disease and endothelial dysfunction (Chen et al., 2008; Li and Kowdley, 2012; Nemecz et al., 2016; Silambarasan et al., 2016). It is plausible that dysregulation of miRNAs may lead to disturbances in the body’s blood pressure regulatory mechanisms and play an important role in the development of HPT. Herein, we investigated circulating levels of miR-1299, miR-30a-5p, miR-30e-3p, miR-126-3p, and miR-182-5p in participants with normal blood pressure, as well as screen-detected and known hypertensives on anti-hypertensive treatment. These miRNAs were chosen as possible targets in our study as next generation sequencing data had previously shown their association with diabetes and HPT (Matsha et al., 2018; Matshazi et al., 2021).

## Materials and Methods

### Design of the Study and Description of Procedures

This study included males and females from the Vascular and Metabolic Health (VMH) study, a sub-study of the Cape Town Bellville South Study (Matsha et al., 2012). The strategies followed with regards to data collection and the conduction of various procedures in this study were previously described by Matsha et al. (2018). In summary, various anthropometric measurements were taken from each participant and for each variable, the average of three separate readings taken was reported. Body Mass Index was calculated as weight per square meter (kg/m^2^) where kg is a participant’s weight in kilograms and m^2^ is the square of their height in meters. World Health Organization guidelines (Chalmers et al., 1999) informed the process of blood pressure measurement. In brief, blood pressure was measured from the right arm of a participant who was in a sitting position and had rested for at least 10-min, using a semi-automatic digital blood pressure monitor (Omron M6 comfort-preformed cuff blood pressure monitor, China). The blood pressure was measured thrice, at three-min intervals, and the lowest systolic blood pressure and corresponding diastolic blood pressure values reported. Participants were then put into their respective blood pressure categories based on; the use of anti-hypertensive medication as known HPT, blood pressure measurement of 140/90 mm Hg and above as screen-detected HPT and normal blood pressure measurement (less than 140/90 mm Hg) as normotensive.

A number of molecular methods were utilized to measure several biochemical parameters in blood samples collected from study participants. These measurements were conducted in an ISO 15189 accredited pathology practice (PathCare Reference Laboratory, Cape Town, South Africa). The molecular methods used included High Performance Liquid Chromatography (BioRad Variant Turbo, BioRad, Hercules, CA, United States), for measurement of glycated hemoglobin, whilst a paramagnetic particle chemiluminescence assay (Beckman DXI, Beckman Coulter, South Africa) was used to measure serum insulin. The Competitive Chemiluminescent (Immulite 2000, Siemens, Munich, Germany) was used to determine levels of cotinine in serum whilst plasma glucose concentration was measured by the enzymatic hexokinase method (Beckman AU, Beckman Coulter, Brea, CA, United States). An enzymatic immunoinhibition—end point assay (Beckman AU, Beckman Coulter, Brea, CA, United States) was used to determine both total cholesterol and high-density lipoprotein cholesterol, whilst triglycerides were measured using a glycerol phosphate oxidase-peroxidase, end point assay (Beckman AU, Beckman Coulter, Brea, CA, United States). The enzymatic selective protection—end point (Beckman AU, Beckman Coulter, Brea, CA, United States) assay was used to determine the levels of low-density lipoprotein cholesterol. In order to determine ultrasensitive C-reactive protein (CRP) levels, a Latex Particle Immunoturbidimetry (Beckman AU, Beckman Coulter, Brea, CA, United States) assay was used. The analysis of miRNA expression through total RNA extraction and quantitative reverse transcription polymerase chain reaction (RT-qPCR) assays was conducted in blood samples that had previously been collected in Tempus Blood RNA tubes (ThermoFisher Scientific, Waltham, MA, United States) and stored at −80°C.

### Total RNA Isolation

The MagMax Total RNA isolation kit (ThermoFisher Scientific, Waltham, MA, United States) was used to extract total RNA (including miRNAs) as per the recommendations of the manufacturer. Total RNA extraction was conducted on thawed, 3 mL whole blood samples. As this was a semi-automated procedure, the Kingfisher Flex system (ThermoFisher Scientific, Waltham, MA, United States) conducted the RNA washing and elution steps. Quality checks were conducted on the extracted nucleic acid by determining the RNA concentration and purity on the NanoDrop One spectrophotometer (ThermoFisher Scientific, Waltham, MA, United States). An extracted total RNA sample with a 260/280 value between 1.8 and 2.0, and whose concentration was greater than 20 ng/μl passed the quality check and was therefore used for downstream applications like RT-qPCR.

### Quantitative Reverse Transcription PCR

In order for miRNA expression to be determined using RT-qPCR, the extracted RNA had to be converted to cDNA first and this conversion was done using the TaqMan Advanced miRNA cDNA synthesis kit (Applied Biosystems, ThermoFisher Scientific, Waltham, MA, United States) as per the manufacturer’s recommendations. In summary, by sequentially conducting poly-A tailing, adaptor ligation, reverse transcription and miR-Amp steps, we converted 2 μl of total RNA into cDNA, which was the starting material in the succeeding PCR step used to determine miRNA expression levels. This PCR analysis was conducted on the QuantStudio 7 Flex real-time PCR instrument (Life Technologies, Carlsbad, CA, United States) using TaqMan miRNA Assay primers (Applied Biosystems, ThermoFisher Scientific, Waltham, MA, United States). The determination of relative miRNA expression in a sample was done using the 2^–Δ*Ct*^, whilst fold change differences in miRNA expression between the study groups were computed with the use of the 2^–ΔΔ*Ct*^ method. In order to normalize miRNA quantification, miR-16-5p (ThermoFisher Scientific, Waltham, MA, United States) was used as the endogenous control (Livak and Schmittgen, 2001).

### Statistical Analysis

The Statistical Product and Service Solutions (SPSS) v.26 software (IBM Corp, United States) was used to conduct data analyses. For normally distributed variables, results are reported as count (and percentages), mean (and standard deviation) whilst for asymmetrically distributed variables, the results are reported as median (25th-75th percentiles). In order to compare the mean and median baseline characteristics across blood pressure groups, the analysis of variance (ANOVA) and Kruskal–Wallis tests were used, respectively. Age, gender and BMI-adjusted Spearman’s partial correlations were used to assess the relationship between miRNAs and other cardiovascular risk profile variables whilst multivariable logistic regression models were used to assess the association of miRNAs with screen-detected and known HPT. A *p*-value less than 0.05 signified statistically significant findings.

## Results

### Study Participant Characteristics

A total of 1,456 participants were included in this study. A summary of the participants’ characteristics is shown in Table 1. There were 386 (67.4%) females and 187 (32.6%) males in the normotensive group with an average age of 39.3 ± 13.7 years, 209 (68.8%) females and 95 (31.2%) males in the screen-detected HPT group with an average age of 48.9 ± 13.4 years whilst the known HPT group comprised of 478 (82.6%) females and 101 (17.4%) males whose average age was 58.9 ± 11.0 years. As expected for HPT, there was a significant difference in the average age of participants across the three groups (*p* < 0.001). Serum gamma-Glutamyltransferase (Gamma GT-S) levels differed significantly between the three groups and the other expected differences (body mass index (BMI), age, systolic blood pressure, waist circumference, high density lipoprotein cholesterol (HDL-c) and low-density lipoprotein (LDL) cholesterol by HPT status in the cardiovascular risk profile were apparent between the participant groups as shown in Table 1.

**TABLE 1:** Study participant characteristics based on blood pressure status.

	Normotensive (*n* = 573) mean ± SD	Screen-detected HPT (*n* = 304) mean ± SD	Known HPT (*n* = 579) mean ± SD	All *p-*value	Normotensive vs Screen-detected HPT *p-*value	Normotensive vs Known HPT *p-*value	Screen-detected HPT vs Known HPT *p-*value
Gender				< 0.001	0.676	< 0.001	<0.001
Female, *n* (%)	386 (67.4)	209 (68.8)	478 (82.6)				
Male, *n* (%)	187 (32.6)	95 (31.2)	101 (17.4)				
Age (years)	39.3 ± 13.7	48.9 ± 13.4	58.9 ± 11.0	< 0.001	<0.001	< 0.001	<0.001
Body mass index (kg/m^2^)	25.9 ± 7.0	28.4 ± 8.5	31.4 ± 7.7	< 0.001	<0.001	< 0.001	<0.001
Waist circumference (cm)	84.5 ± 15.3	91.2 ± 16.3	98.5 ± 16.4	< 0.001	<0.001	< 0.001	<0.001
Hip circumference (cm)	98.2 ± 15.0	102.1 ± 16.4	108.5 ± 16.2	< 0.001	<0.001	< 0.001	<0.001
Waist to Hip ratio	0.9 ± 0.1	0.9 ± 0.1	0.9 ± 0.1	< 0.001	<0.001	< 0.001	0.006
Systolic blood pressure (mmHg)	115.5 ± 12.7	151.7 ± 19.5	148.2 ± 26.4	< 0.001	<0.001	< 0.001	0.041
Diastolic blood pressure (mmHg)	74.7 ± 9.1	95.8 ± 11.6	90.7 ± 15.4	< 0.001	<0.001	< 0.001	<0.001
Fasting Blood glucose(mmol/L)*	4.6 (4.3; 5.0)	4.9 (4.6; 5.5)	5.3 (4.9; 6.8)	< 0.001	<0.001	< 0.001	<0.001
2-h glucose (mmol/L)*	5.3 (4.3; 6.6)	6.1 (5.0; 7.7)	6.9 (5.5; 8.6)	< 0.001	<0.001	< 0.001	<0.001
HbA1c (%)	5.7 ± 1.0	5.9 ± 1.3	6.8 ± 1.9	0.032	0.006	< 0.001	<0.001
HbA1c (mmol/mol)	38.6 ± 11.0	40.9 ± 13.8	50.7 ± 21.1	0.032	0.006	< 0.001	<0.001
Fasting Insulin (mIU/L)*	5.6 (3.4; 8.8)	6.1 (3.9; 9.6)	8.0 (5.0; 13.1)	< 0.001	0.065	< 0.001	<0.001
2-h Insulin (mIU/L)*	28.8 (15.0; 53.4)	34.7 (19.1; 64.0)	48.7 (26.5; 88.2)	< 0.001	0.014	< 0.001	<0.001
Triglycerides (mmol/L)*	1.0 (0.7; 1.4)	1.2 (0.9; 1.7)	1.4 (1.0; 1.9)	< 0.001	<0.001	< 0.001	<0.001
Total Cholesterol (mmol/L)	4.8 ± 1.1	5.2 ± 1.1	5.4 ± 1.2	< 0.001	<0.001	< 0.001	<0.001
HDL-cholesterol (mmol/L)	1.3 ± 0.4	1.4 ± 0.5	1.3 ± 0.3	< 0.001	<0.001	0.359	< 0.001
LDL-cholesterol (mmol/L)	3.0 ± 1.0	3.2 ± 1.0	3.3 ± 1.0	< 0.001	0.025	< 0.001	0.021
C-Reactive Protein (mg/L)*	2.7 (1.1; 6.9)	3.7 (1.5; 9.3)	5.2 (2.5; 10.1)	< 0.001	0.012	< 0.001	<0.001
Gamma GT (IU/L)*	25 (18; 39)	30 (21; 50)	32 (22; 53)	< 0.001	<0.001	< 0.001	<0.001
Serum Creatinine (μmol/L)	59.5 ± 12.0	62.7 ± 28.9	71.8 ± 61.0	< 0.001	0.021	< 0.001	0.014

Values presented as mean ± SD unless marked with an asterisk*, in which case the median and (25th–75th percentiles) are reported.

*The Kruskal–Wallis test and analysis of variance (ANOVA) were used to compare the median and mean baseline characteristics, respectively, across blood pressure groups.*

*All *p-value* shows a comparison of the three blood pressure groups together (i.e., normotensives vs screen-detected HPT vs known HPT).*

*HPT, hypertension; SD, standard deviation; HbA1c, glycated hemoglobin.*

### Relative MicroRNA Expression

**FIGURE 1:**
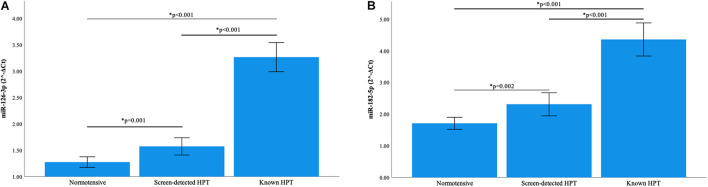
Comparison of the relative expression of miR-126-3p and 182-5p in the three blood pressure groups. **(A)** The expression of miR-126-3p was significantly higher in known hypertensives when compared to screen-detected hypertensives (*p* < 0.001) and the normotensives (*p* < 0.001). There was also a significant difference in expression when screen-detected hypertensives were compared to the normotensives (*p* = 0.001). **(B)** There was a significantly higher expression of miR-182-5p in known hypertensives when compared to screen-detected hypertensives (*p* < 0.001) and the normotensives (*p* < 0.001). When screen-detected hypertensives were compared to the normotensives, that significant difference in expression remained (*p* = 0.002). The relative miRNA expressions were calculated using the 2^– Δ*Ct*^ method and a one-way ANOVA used to compare differences in miRNA expression between groups. The symbol * denotes a statistically significant *p*-value.

**FIGURE 2:**
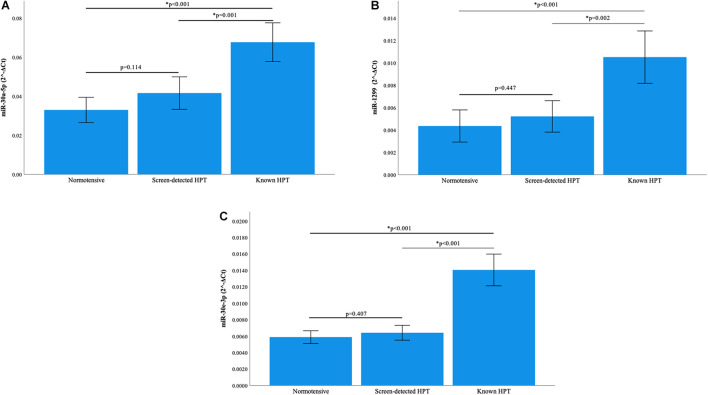
Comparison of the relative expression of miR-30a-5p, miR-1299 and miR-30e-3p. **(A)** The expression of the miR-30a-5p was significantly higher in known hypertension compared to screen-detected hypertension (*p* = 0.001) and the normotensives (*p* < 0.001). However, there was no significant difference in expression between screen-detected hypertensives and normotensives (*p* = 0.114). **(B)** There was a significantly higher expression of miR-1299 in known hypertension when compared to screen-detected hypertension (*p* = 0.002) and the normotensives (*p* < 0.001). There was no significant difference in expression when screen-detected hypertensives were compared to the normotensives (*p* = 0.447). **(C)** The expression of miR-30e-3p was significantly higher in known hypertension when compared to screen-detected hypertension (*p* < 0.001) and the normotensives (*p* < 0.001). There was no significant difference in expression when screen-detected hypertensives were compared to the normotensives (*p* = 0.407). The relative miRNA expressions were calculated using the 2^– Δ*Ct*^ method and a one-way ANOVA used to compare differences in miRNA expression between groups. The symbol * denotes a statistically significant *p*-value.

Normalization of relative miRNA expression was done with reference to the expression of miR-16-5p. Overall, the expression of miR-126-3p and 182-5p differed significantly across all blood pressure groups. Figure 1A shows that in participants with known HPT, the relative expression (2^–Δ*Ct*^) of miR-126-3p (3.266) was significantly higher than that of the normotensives (1.275) and screen-detected hypertensives (1.573), *p* < 0.001). For miR-182-5p, the relative expression in known HPT was significantly higher (4.360) compared to both normotensives (1.711) and screen detected hypertensives (2.312), both *p* < 0.001 as shown in Figure 1B. However, for miR-30a-5p, 1299 and 30e-3p, whilst there were significant differences in their relative expression (2^–Δ*Ct*^) in known HPT (0.068; 0.011 and 0.014) vs normotensives (0.033; 0.004 and 0.006), respectively, (all *p* < 0.001), that significance was not seen when screen-detected hypertensives were compared to the normotensives, all *p* ≥ 0.114 as shown in Figures 2A–C.

### Fold Change Computation

When compared to the normotensives, there was at least a 2.2-fold increase in expression of miR-126-3p, 182-5p, 30a-5p, 1299, and 30e-3p in the known HPT group. MiR-126-3p was the most expressed of the five miRNAs, particularly in known hypertensives versus normotensives (fold change = 2.64) and known hypertensives versus screen-detected hypertensives (fold change = 1.99). However, the fold difference in expression of all five miRNAs in the screen-detected hypertensive participants compared to the normotensives was ≤ 1.65-fold, with the lowest fold difference in expression between these two blood pressure groups observed for miR-30e-3p, whose expression was 1.17-fold higher in screen-detected hypertensives compared to the normotensives.

### Correlation of MicroRNA Expression With Anthropometric Measurements

There was a significant positive correlation between the expression of the five miRNAs across the blood pressure groups (*r* ≥ 0.71, *p* < 0.001), with the highest correlation in expression seen between miR-126-3p and miR-182-5p (*r* = 0.983, *p* < 0.001) as shown in Table 2. Detailed correlations between the expression of each miRNA and biochemical parameters are shown in the Supplementary Tables (see Supplementary Tables 1–5). The expression of all miRNAs correlated negatively with waist circumference, with the highest correlation coefficients seen in the screen-detected HPT group for both miR-126-3p (*r* = −0.748, *p* < 0.001) and miR-30e-3p (*r* = −0.729, *p* < 0.001). There was a significant, though weak correlation between the expression of both miR-182-5p and miR-30a-5p with systolic blood pressure in the known HPT group. However, there was no significant association between the expression of miR-30e-3p, miR-126-3p and 1299 with systolic blood pressure regardless of blood pressure status. Whilst there was no correlation between the expression of any of the five miRNAs and total cholesterol (TC), there was a significant positive correlation with HDL-c, with the highest correlation coefficients seen in the known HPT group with respect to miR-182-5p (*r* = 0.629, *p* = 0.001), miR-30a-5p (*r* = 0.615, *p* = 0.002), miR-30e-3p (*r* = 0.608, *p* = 0.002) miR-126-3p (*r* = 0.595, *p* = 0.003) and finally miR-1299 (*r* = 0.508, *p* = 0.013). Gamma GT-S also showed a significantly positive correlation with the expression of all but miR-1299 in the screen-detected HPT group.

**TABLE 2:** Partial correlations between miRNA relative expression and anthropometric and biochemical parameters.

	miR-30a-5p 2^–Δ*Ct*^		miR-126-3p 2^–Δ*Ct*^		miR-182-5p 2^–Δ*Ct*^		miR-30e-3p 2^–Δ*Ct*^		miR-1299 2^–Δ*Ct*^	
					
	r	p-value	r	p-value	r	p-value	r	p-value	r	p-value
miR-30a-5p 2^–Δ*Ct*^	1.000		0.901	< 0.001	0.937	< 0.001	0.901	< 0.001	0.710	< 0.001
miR-1299 2^–Δ*Ct*^	0.710	< 0.001	0.731	< 0.001	0.738	< 0.001	0.721	< 0.001	1.000	
miR-182-5p 2^–Δ*Ct*^	0.937	< 0.001	0.983	< 0.001	1.000		0.968	< 0.001	0.738	< 0.001
miR-30e-3p 2^–Δ*Ct*^	0.901	< 0.001	0.973	< 0.001	0.968	< 0.001	1.000		0.721	< 0.001
miR-126-3p 2^–Δ*Ct*^	0.901	< 0.001	1.000		0.983	< 0.001	0.973	< 0.001	0.731	< 0.001
Waist circumference (cm)	−0.485	0.019	−0.535	0.009	−0.518	0.011	−0.538	0.008	−0.521	0.011
Hip circumference (cm)	−0.154	0.482	−0.053	0.809	−0.100	0.651	−0.084	0.703	0.048	0.828
Waist to Hip ratio	0.001	0.997	−0.113	0.608	−0.060	0.787	−0.083	0.705	−0.131	0.553
Systolic Blood Pressure (mmHg)	0.313	0.146	0.293	0.174	0.304	0.158	0.275	0.204	0.268	0.216
Diastolic Blood Pressure (mmHg)	0.224	0.304	0.217	0.319	0.228	0.295	0.202	0.354	0.233	0.284
Fasting Blood Glucose (mmol/L)	0.133	0.546	0.077	0.728	0.109	0.621	0.119	0.590	0.040	0.856
2-h glucose (mmol/L)	0.046	0.834	0.019	0.932	0.044	0.841	0.058	0.792	0.102	0.644
HbA1c (%)	−0.201	0.357	−0.170	0.439	−0.190	0.385	−0.153	0.485	−0.130	0.555
Fasting insulin (mIU/L)	0.272	0.209	0.241	0.267	0.267	0.218	0.250	0.250	0.216	0.322
2-h insulin (mIU/L)	0.049	0.826	0.024	0.913	0.051	0.815	0.045	0.838	0.078	0.723
Triglycerides-S (mmol/L)	0.037	0.868	0.018	0.935	0.008	0.971	−0.024	0.915	−0.079	0.719
Total cholesterol (mmol/L)	0.108	0.624	0.019	0.930	0.039	0.858	0.015	0.945	0.041	0.854
HDL-cholesterol (mmol/L)	0.445	0.033	0.401	0.058	0.431	0.040	0.443	0.034	0.421	0.045
LDL-cholesterol (mmol/L)	0.065	0.769	−0.017	0.939	−0.003	0.991	−0.025	0.908	0.021	0.925
C-Reactive Protein (mg/L)	0.145	0.509	0.139	0.528	0.144	0.511	0.173	0.429	0.152	0.487
Gamma GT (IU/L)	0.094	0.669	0.047	0.830	0.078	0.722	0.063	0.775	0.043	0.845
Serum creatinine (μmol/L)	−0.349	0.443	−0.390	0.387	−0.377	0.404	−0.385	0.394	−0.337	0.460

*Age, gender, and BMI adjusted Spearman’s partial correlations were used to assess the association between miRNA relative expression and anthropometric and biochemical parameters across the three blood pressure groups.*

### Multivariable Regression Analysis

The results of multivariable regression analysis are shown in Table 3. With regards to miR-126-3p, the crude odds (age and gender adjusted only) ratio (OR) was 1.16 (95% confidence interval (CI): 1.05-1.27, ‘*p* = 0.003) for screen-detected HPT, whilst for known HPT, the OR was 1.58 (95% CI: 1.46-1.71, *p* < 0.001). For miR-30a-5p, the OR was 1.24 (95% CI: 1.00-1.55, *p* = 0.053) for screen-detected HPT whilst for known HPT, the OR was 1.63 (95% CI: 1.36-1.95, *p* < 0.001). The crude odds ratio for miR-182-5p was 1.11 (95% CI: 1.05-1.17, *p* < 0.001) for screen-detected HPT whilst for known HPT, the OR was 1.24 (95% CI: 1.18-1.30, *p* < 0.001). The associations between the expression of these miR-126-3p, 182-5p and 30a-5p and HPT (both screen-detected and known) remained significant even when the model was adjusted for BMI, TC, triglycerides (TG) and glycated hemoglobin (HbA1c). When further adjusted for duration of known HPT diagnosis, the associations also remained significant for these three miRNAs. As for miR-30e-3p, the only significant association was with known HPT which had an OR of 1.71 (95% CI: 1.46–2.01, *p* < 0.001) whilst for screen-detected HPT, the OR was 1.07 (95% CI: 0.91–1.26, *p* = 0.393). There was no significant association between the expression of miR-1299 and screen-detected or known HPT.

**TABLE 3:** Multivariable regression analysis of miRNAs for the presence of screen-detected and known hypertension.

	Screen-detected HPT			Known HPT		
		
	OR	95% CI	*p-value*	OR	95% CI	*p-value*
miR 30a-5p**						
Model 1	1.24	(1.00; 1.55)	0.053	1.63	(1.36;1.95)	< 0.001
Model 2	1.35	(1.07; 1.72)	0.013	1.98	(1.58;2.48)	< 0.001
Model 3	1.35	(1.06; 1.71)	0.015	1.97	(1.57;2.48)	< 0.001
Model 4	1.37	(1.07; 1.74)	0.011	1.99	(1.57;2.51)	< 0.001
Model 5	1.35	(1.06; 1.72)	0.014	1.97	(1.56;2.48)	< 0.001
Model 6	–	–	–	1.81	(1.4;2.34)	< 0.001
miR 30e-3p***						
Model 1	1.07	(0.91; 1.26)	0.393	1.58	(1.4;1.78)	< 0.001
Model 2	1.14	(0.96; 1.36)	0.130	1.71	(1.46;1.99)	< 0.001
Model 3	1.15	(0.96; 1.37)	0.121	1.71	(1.46;2.01)	< 0.001
Model 4	1.16	(0.97; 1.39)	0.094	1.74	(1.48;2.05)	< 0.001
Model 5	1.17	(0.98; 1.39)	0.089	1.74	(1.48;2.05)	< 0.001
Model 6	–	–	–	1.81	(1.42;2.30)	< 0.001
miR 126-3p*						
Model 1	1.16	(1.05; 1.27)	0.003	1.58	(1.46;1.71)	< 0.001
Model 2	1.23	(1.11; 1.37)	< 0.001	1.72	(1.55;1.9)	< 0.001
Model 3	1.21	(1.09; 1.35)	< 0.001	1.69	(1.52;1.87)	< 0.001
Model 4	1.23	(1.11; 1.38)	< 0.001	1.71	(1.54;1.91)	< 0.001
Model 5	1.23	(1.1; 1.37)	< 0.001	1.71	(1.53;1.91)	< 0.001
Model 6	–	–	–	1.93	(1.65;2.26)	< 0.001
miR 1299**						
Model 1	1.07	(0.94; 1.21)	0.329	1.22	(1.11;1.35)	< 0.001
Model 2	1.59	(0.42; 5.96)	0.495	1.18	(1.05;1.33)	0.005
Model 3	1.05	(0.92; 1.2)	0.466	1.19	(1.05;1.34)	0.006
Model 4	1.08	(0.94; 1.24)	0.308	1.21	(1.06;1.37)	0.005
Model 5	1.09	(0.94; 1.25)	0.254	1.22	(1.07;1.39)	0.004
Model 6	–	–	–	1.23	(1.05;1.44)	0.009
miR 182-5p*						
Model 1	1.11	(1.05; 1.17)	< 0.001	1.24	(1.18;1.3)	< 0.001
Model 2	1.14	(1.07; 1.21)	< 0.001	1.31	(1.24;1.39)	< 0.001
Model 3	1.14	(1.07; 1.21)	< 0.001	1.31	(1.23;1.39)	< 0.001
Model 4	1.14	(1.08; 1.22)	< 0.001	1.31	(1.23;1.4)	< 0.001
Model 5	1.14	(1.07; 1.22)	< 0.001	1.31	(1.23;1.39)	< 0.001
Model 6	–	–	–	1.31	(1.21;1.41)	< 0.001

Model 1: Crude; Model 2: included age and sex; Model 3: included age, sex and BMI; Model 4: included age, sex, BMI, HbA1c; Model 5: included age, sex, BMI, HbA1c, triglycerides, total cholesterol; Model 6: included age, sex, BMI, HbA1c, triglycerides, total cholesterol; duration of disease *calculated for 0.1-unit increase; ** calculated for 0.01-unit increase; ***calculated for 0.001-unit increase.

## Discussion

This study demonstrated a significantly higher expression of miR-126-3p and miR-182-5p in hypertensives (both screen-detected and known) when compared to the normotensives. However, there was no significant difference in the expression levels of miR-30a-5p, 30e-3p, and 1299 in normotensives, relative to screen-detected hypertensives. Although multivariable logistic regressions showed no association between HPT and the expression of both miR-30e-3p and miR-1299, we observed an association between the expression of miR-126-3p, 182-5p and 30a-5p with screen-detected and known HPT, particularly in the latter. Even after adjustment of the crude model for age, sex, BMI, HbA1c, TG, and TC, the associations remained significant.

Angiogenesis and maintenance of vascular integrity are vital blood pressure regulatory processes in which miR-126 has an essential role (Wang et al., 2008). During hypertensive states, there is loss of endothelial cell function and blood perfusion to the capillaries becomes limited, leading to capillary disappearance. This is described as microvascular rarefaction and is a distinctive characteristic of HPT (Humar et al., 2009). Its effects contribute to HPT-related complications such as organ damage and stroke. It has been reported that miR-126, through the stimulation of proangiogenic activity of vascular endothelial growth factor (VEGF) and fibroblast growth factor (FGF), counteracts microvascular rarefaction by encouraging the formation of blood vessels. This is achieved through the quelling of Spred-1 expression, a known inhibitor of angiogenic signaling (Wang et al., 2008). Our study demonstrated a higher expression of miR-126-3p in hypertensive participants when compared to the normotensives, and this finding has also been reported in another study (Liu et al., 2018). It is plausible that the high expression of miR-126 in hypertensives represents a response to lower the blood pressure through promotion of blood vessel formation, whilst repressing the effects of anti-angiogenic Spred-1. Whilst our study demonstrated a higher expression of miR-126-3p in the whole blood of hypertensives compared to the normotensives, another study demonstrated lower expression of miR-126 in hypertensives when compared to healthy controls, albeit in peripheral blood mononuclear cells (PBMCs) (Kontaraki et al., 2014). There was no significant difference in miR-126 expression between hypertensive and normotensive participants in the study by Chen and co-workers (Chen et al., 2018). These discrepancies could in part be explained by the different sample types and participant recruitment criteria used. It has also been demonstrated that anti-hypertensive medications like nebivolol and atenolol affect miRNA expression (Ye et al., 2013). Kontaraki and colleagues excluded anyone on anti-hypertensive medication from their study, while Chen and colleagues’ hypertensive group included any participants who had been on anti-hypertensive therapy for longer than 3 months (Chen et al., 2018).

The efficacy of miR-182 against glioblastoma multiforme, a therapy-resistant cancer of the brain has been previously reported (Kouri et al., 2015). It has been established as an oncogenic miRNA and its interactions in several types of cancers have been reviewed (Wei et al., 2015). The expression of miR-182-5p was significantly higher in both hypertensive groups when compared to the normotensives in our study. However, there is a paucity of studies indicating a role for miR-182-5p in essential HPT. Nonetheless, in a study evaluating the miRNA expression profiles in the placenta of pregnant participants, the expression of miR-182 was found to be elevated in participants with pre-eclampsia, a mid-term complication of pregnancy characterized by proteinuria and HPT, when compared to normal, pregnant controls (Pineles et al., 2007). Hypertension is a known risk factor for coronary artery disease (CAD) (Weber et al., 2016) and in participants with unprotected left main CAD, the plasma expression of miR-182-5p was significantly higher compared to the non-CAD control group and further analyses indicated its high diagnostic power for uncontrolled left main CAD (Zhu et al., 2019). Whether miR-182-5p plays a role in hemodynamic regulation or indeed the pathogenesis of HPT remains to be elucidated.

In our study, the expression of miR-30a-5p was significantly higher in known hypertensives compared to normotensives. The upregulation of miR-30a in hypertensive participants compared to normal controls was also reported in another study and identified as a possible biomarker target for differentiating white coat HPT from essential HPT and normotensives (Huang et al., 2016). Delta-like ligand 4 (dll4) is mainly expressed in the vascular endothelium and exerts its effects through Notch signaling by attachment to its receptor Notch1. The ligand is a key inhibitor of angiogenesis and the expression of miR-30a works against the expression of dll4 and in doing so, promotes angiogenesis (Jiang et al., 2013). The promotion of angiogenesis through the expression of miR-30a-5p can lead to lowering of the blood pressure and could have been the case in our cohort of known hypertensives.

A limitation of our study was that participants making up the three blood pressure groups were not age and gender-matched. Although these variables were adjusted for in the multivariable regression analysis, their effect may not have been completely eliminated. However, the overall large number of participants involved in the project provided sufficient power to the study as each blood pressure group was adequately represented. Whilst the cross-sectional nature of the study precludes inference about causal relationships between miRNA expression and development of HPT, it provided a basis for setting up longitudinal cohorts in which functional studies can be conducted to further clarify the roles that these non-coding ribonucleic acids (RNAs) play in the pathogenesis of HPT.

In conclusion, we demonstrated for the first time in an African population, the differential expression of miRNAs in the whole blood of participants on the basis of their blood pressure status. These miRNAs could form a panel of biomarker targets for HPT diagnostic purposes. Furthermore, the study validated prior findings on miR-126 and the miR-30 family and highlighted their possible involvement in the pathogenesis of HPT, warranting further investigation into these non-coding RNAs as they could offer potential prognostic and therapeutic avenues for cardiovascular diseases.

## Data Availability Statement

Some of the datasets used and/or analyzed during the current study are available from the National Centre for Biotechnology Information (NCBI) Sequence Read Archive on the following link: https://www.ncbi.nlm.nih.gov/sra/PRJNA680302. Other datasets presented are not readily available because of the terms of consent to which participants agreed, but are available from the principal investigator of the main study on reasonable request, which should be directed to TM, matshat@cput.ac.za.

## Ethics Statement

This investigation was based on the Cape Town Vascular and Metabolic Health (VMH) study, which was approved by the Research Ethics Committees of the Cape Peninsula University of Technology (CPUT) and Stellenbosch University (respectively, NHREC: REC — 230 408 — 014 and N14/01/003). Ethical approval was also obtained for this cross-sectional sub-study from the CPUT Health and Wellness Sciences Research Ethics Committee (CPUT/HW-REC 2019/H7). The study was conducted as per the provisions of the Declaration of Helsinki. All procedures were explained to the participants in their language of choice. Once the participants fully understood their participation, they signed informed consent forms to allow the collection of blood and anthropometric data.

## Author Contributions

TM, RE, and AK: conceptualization and funding acquisition. DM, SR, and CW: methodology. DM and SD: formal analysis. DM and CW: investigation. TM: resources. DM, SR, and SD: data curation. DM: writing—original draft preparation. TM, RE, AK, GD, and SR: writing—review and editing. DM, CW, and SR: validation. DM, SD, and TM: visualization. TM and GD: supervision. TM and SD: project administration. All authors have read and agreed to the published version of the manuscript.

## Conflict of Interest

The authors declare that the research was conducted in the absence of any commercial or financial relationships that could be construed as a potential conflict of interest.

## Publisher’s Note

All claims expressed in this article are solely those of the authors and do not necessarily represent those of their affiliated organizations, or those of the publisher, the editors and the reviewers. Any product that may be evaluated in this article, or claim that may be made by its manufacturer, is not guaranteed or endorsed by the publisher.
