# Supplementary material for: Circulating Levels of MicroRNAs Associated With Hypertension: A Cross-Sectional Study in Male and Female South African Participants
Source: Front Genet. 2021 Sep 14;12:710438. doi: 10.3389/fgene.2021.710438 (PMC8476992; doi:10.3389/fgene.2021.710438)
Supplement: Supplementary file 1 [file Data_Sheet_1.docx]

Supplementary Material

# Supplementary Tables

Tables S1-5 show data on Spearman correlation co-efficient analysis. The comparisons show the relationship between miRNA expression and anthropometric data within each of the blood pressure groups.

Table S1. Age, gender and BMI adjusted partial correlation coefficients for the association between miR-30a-5p relative expression and anthropometric and biochemical parameters according to blood pressure status

|  | **All** | | **Normotensive** | | **Screen-detected HPT** | | **Known HPT** | |
| --- | --- | --- | --- | --- | --- | --- | --- | --- |
|  | r | *p-value* | r | *p-value* | r | *p-value* | r | *p-value* |
| miR-30a-5p 2^-ΔCt^ | 1.000 |  | 1.000 |  | 1.000 |  | 1.000 |  |
| miR-1299 2^-ΔCt^ | 0.710 | <0.001 | 0.643 | 0.001 | 0.734 | <0.001 | 0.774 | <0.001 |
| miR-182-5p 2^-ΔCt^ | 0.937 | <0.001 | 0.934 | <0.001 | 0.923 | <0.001 | 0.945 | <0.001 |
| miR-30e-3p 2^-ΔCt^ | 0.901 | <0.001 | 0.880 | <0.001 | 0.867 | <0.001 | 0.934 | <0.001 |
| miR-126-3p 2^-ΔCt^ | 0.901 | <0.001 | 0.891 | <0.001 | 0.878 | <0.001 | 0.915 | <0.001 |
| Waist circumference(cm) | -0.485 | 0.019 | -0.518 | 0.011 | -0.602 | 0.002 | -0.416 | 0.048 |
| Hip circumference (cm) | -0.154 | 0.482 | -0.048 | 0.827 | -0.278 | 0.199 | -0.022 | 0.921 |
| Waist to Hip ratio | 0.001 | 0.997 | 0.024 | 0.913 | 0.127 | 0.564 | -0.063 | 0.774 |
| Systolic blood pressure (mmHg) | 0.313 | 0.146 | -0.019 | 0.931 | 0.376 | 0.077 | 0.448 | 0.032 |
| Diastolic blood pressure (mmHg) | 0.224 | 0.304 | 0.051 | 0.817 | 0.239 | 0.272 | 0.309 | 0.152 |
| Fasting Blood glucose (mmol/L) | 0.133 | 0.546 | 0.253 | 0.244 | 0.238 | 0.275 | 0.012 | 0.957 |
| 2-hour glucose (mmol/L) | 0.046 | 0.834 | 0.202 | 0.355 | 0.244 | 0.262 | -0.123 | 0.576 |
| HbA1c (%) | -0.201 | 0.357 | -0.151 | 0.492 | -0.066 | 0.763 | -0.197 | 0.368 |
| Fasting insulin (mIU/L) | 0.272 | 0.209 | 0.418 | 0.047 | 0.185 | 0.397 | 0.075 | 0.734 |
| 2-hour insulin (mIU/L) | 0.049 | 0.826 | 0.222 | 0.308 | 0.001 | 0.997 | -0.053 | 0.811 |
| Triglycerides-S (mmol/L) | 0.037 | 0.868 | 0.079 | 0.721 | 0.028 | 0.900 | 0.089 | 0.686 |
| Total cholesterol (mmol/L) | 0.108 | 0.624 | 0.140 | 0.524 | 0.342 | 0.110 | 0.255 | 0.240 |
| HDL-cholesterol (mmol/L) | 0.445 | 0.033 | 0.417 | 0.048 | 0.565 | 0.005 | 0.615 | 0.002 |
| LDL-cholesterol (mmol/L) | 0.065 | 0.769 | 0.128 | 0.561 | 0.284 | 0.189 | 0.177 | 0.419 |
| C-Reactive Protein (mg/L) | 0.145 | 0.509 | 0.109 | 0.619 | 0.184 | 0.402 | 0.255 | 0.240 |
| Gamma GT (IU/L) | 0.094 | 0.669 | -0.068 | 0.758 | 0.471 | 0.023 | 0.182 | 0.407 |
| S-Creatinine (µmol/L) | -0.349 | 0.443 | -0.283 | 0.539 | -0.472 | 0.285 | -0.242 | 0.601 |
| Duration of disease (years) | - | - | - | - | - | - | 0.358 | 0.430 |

**Table S2.** Age, gender and BMI adjusted partial correlation coefficients for the association between miR-1299 relative expression and anthropometric and biochemical parameters according to blood pressure status

|  | **All** | | **Normotensive** | | **Screen-detected HPT** | | **Known HPT** | |
| --- | --- | --- | --- | --- | --- | --- | --- | --- |
|  | r | *p-value* | r | *p-value* | r | *p-value* | r | *p-value* |
| miR-30a-5p 2^-ΔCt^ | 0.710 | <0.001 | 0.643 | 0.001 | 0.734 | <0.001 | 0.774 | <0.001 |
| miR-1299 2^-ΔCt^ | 1.000 |  | 1.000 |  | 1.000 |  | 1.000 |  |
| miR-182-5p 2^-ΔCt^ | 0.738 | <0.001 | 0.708 | <0.001 | 0.692 | <0.001 | 0.795 | <0.001 |
| miR-30e-3p 2^-ΔCt^ | 0.721 | <0.001 | 0.678 | <0.001 | 0.690 | <0.001 | 0.796 | <0.001 |
| miR-126-3p 2^-ΔCt^ | 0.731 | <0.001 | 0.678 | <0.001 | 0.697 | <0.001 | 0.807 | <0.001 |
| Waist circumference (cm) | -0.521 | 0.011 | -0.513 | 0.012 | -0.493 | 0.017 | -0.526 | 0.010 |
| Hip circumference (cm) | 0.048 | 0.828 | 0.213 | 0.330 | -0.015 | 0.945 | 0.048 | 0.828 |
| Waist to Hip ratio | -0.131 | 0.553 | -0.154 | 0.482 | 0.073 | 0.742 | -0.222 | 0.309 |
| Systolic blood pressure (mmHg) | 0.268 | 0.216 | 0.014 | 0.948 | 0.362 | 0.089 | 0.367 | 0.085 |
| Diastolic blood pressure (mmHg) | 0.233 | 0.284 | 0.143 | 0.516 | 0.347 | 0.105 | 0.259 | 0.233 |
| Fasting Blood glucose (mmol/L) | 0.040 | 0.856 | 0.144 | 0.512 | 0.123 | 0.575 | -0.056 | 0.799 |
| 2-hour glucose (mmol/L) | 0.102 | 0.644 | 0.207 | 0.342 | 0.362 | 0.090 | -0.078 | 0.724 |
| HbA1c (%) | -0.130 | 0.555 | 0.031 | 0.887 | -0.146 | 0.506 | -0.158 | 0.473 |
| Fasting insulin (mIU/L) | 0.216 | 0.322 | 0.367 | 0.085 | 0.088 | 0.689 | 0.039 | 0.860 |
| 2-hour insulin (mIU/L) | 0.078 | 0.723 | 0.215 | 0.324 | 0.104 | 0.637 | -0.039 | 0.860 |
| Triglycerides-S (mmol/L) | -0.079 | 0.719 | -0.047 | 0.830 | -0.116 | 0.598 | 0.017 | 0.937 |
| Total cholesterol (mmol/L) | 0.041 | 0.854 | 0.058 | 0.794 | 0.250 | 0.250 | 0.220 | 0.313 |
| HDL-cholesterol (mmol/L) | 0.421 | 0.045 | 0.502 | 0.015 | 0.527 | 0.010 | 0.508 | 0.013 |
| LDL-cholesterol (mmol/L) | 0.021 | 0.925 | 0.035 | 0.874 | 0.210 | 0.335 | 0.188 | 0.392 |
| C-Reactive Protein (mg/L) | 0.152 | 0.487 | 0.165 | 0.453 | 0.233 | 0.284 | 0.201 | 0.358 |
| Gamma GT (IU/L) | 0.043 | 0.845 | 0.058 | 0.792 | 0.299 | 0.166 | 0.063 | 0.776 |
| S-Creatinine (µmol/L) | -0.337 | 0.460 | -0.298 | 0.517 | -0.441 | 0.322 | -0.247 | 0.593 |
| Duration of disease (years) | - | - | - | - | - | - | 0.202 | 0.664 |

**Table S3.** Age, gender and BMI adjusted partial correlation coefficients for the association between miR-182-5p relative expression and anthropometric and biochemical parameters according to blood pressure status

|  | **All** | | **Normotensive** | | **Screen-detected HPT** | | **Known HPT** | |
| --- | --- | --- | --- | --- | --- | --- | --- | --- |
|  | r | *p-value* | r | *p-value* | r | *p-value* | r | *p-value* |
| miR-30a-5p 2^-ΔCt^ | 0.937 | <0.001 | 0.934 | <0.001 | 0.923 | <0.001 | 0.945 | <0.001 |
| miR-1299 2^-ΔCt^ | 0.738 | <0.001 | 0.708 | <0.001 | 0.692 | <0.001 | 0.795 | <0.001 |
| miR-182-5p 2^-ΔCt^ | 1.000 |  | 1.000 |  | 1.000 |  | 1.000 |  |
| miR-30e-3p 2^-ΔCt^ | 0.968 | <0.001 | 0.961 | <0.001 | 0.949 | <0.001 | 0.980 | <0.001 |
| miR-126-3p 2^-ΔCt^ | 0.983 | <0.001 | 0.974 | <0.001 | 0.968 | <0.001 | 0.982 | <0.001 |
| Waist circumference(cm) | -0.518 | 0.011 | -0.547 | 0.007 | -0.685 | <0.001 | -0.453 | 0.030 |
| Hip circumference (cm) | -0.100 | 0.651 | 0.051 | 0.816 | -0.218 | 0.318 | 0.011 | 0.959 |
| Waist to Hip ratio | -0.060 | 0.787 | -0.048 | 0.826 | 0.036 | 0.870 | -0.133 | 0.544 |
| Systolic blood pressure (mmHg) | 0.304 | 0.158 | -0.021 | 0.923 | 0.327 | 0.128 | 0.415 | 0.049 |
| Diastolic blood pressure (mmHg) | 0.228 | 0.295 | 0.095 | 0.667 | 0.233 | 0.285 | 0.284 | 0.189 |
| Fasting Blood glucose (mmol/L) | 0.109 | 0.621 | 0.191 | 0.384 | 0.239 | 0.273 | 0.006 | 0.980 |
| 2-hour glucose (mmol/L) | 0.044 | 0.841 | 0.184 | 0.402 | 0.248 | 0.253 | -0.131 | 0.550 |
| HbA1c (%) | -0.190 | 0.385 | -0.175 | 0.426 | 0.021 | 0.924 | -0.215 | 0.325 |
| Fasting insulin (mIU/L) | 0.267 | 0.218 | 0.422 | 0.045 | 0.220 | 0.314 | 0.041 | 0.853 |
| 2-hour insulin (mIU/L) | 0.051 | 0.815 | 0.230 | 0.291 | 0.039 | 0.860 | -0.081 | 0.714 |
| Triglycerides-S (mmol/L) | 0.008 | 0.971 | 0.018 | 0.935 | 0.134 | 0.541 | 0.013 | 0.952 |
| Total cholesterol (mmol/L) | 0.039 | 0.858 | 0.097 | 0.661 | 0.235 | 0.281 | 0.220 | 0.312 |
| HDL-cholesterol (mmol/L) | 0.431 | 0.040 | 0.459 | 0.028 | 0.486 | 0.019 | 0.629 | 0.001 |
| LDL-cholesterol (mmol/L) | -0.003 | 0.991 | 0.077 | 0.728 | 0.185 | 0.398 | 0.146 | 0.508 |
| C-Reactive Protein (mg/L) | 0.144 | 0.511 | 0.169 | 0.442 | 0.154 | 0.484 | 0.157 | 0.474 |
| Gamma GT (IU/L) | 0.078 | 0.722 | -0.052 | 0.814 | 0.472 | 0.023 | 0.143 | 0.514 |
| S-Creatinine (µmol/L) | -0.377 | 0.404 | -0.335 | 0.463 | -0.503 | 0.249 | -0.268 | 0.561 |
| Duration of disease (years) | - | - | - | - | - | - | 0.318 | 0.487 |

**Table** **S4.** Age, gender and BMI adjusted partial correlation coefficients for the association between miR-30e-3p relative expression and anthropometric and biochemical parameters according to blood pressure status

|  | **All** | | **Normotensive** | | **Screen-detected HPT** | | **Known HPT** | |
| --- | --- | --- | --- | --- | --- | --- | --- | --- |
|  | r | *p-value* | r | *p-value* | r | *p-value* | r | *p-value* |
| miR-30a-5p 2^-ΔCt^ | 0.901 | <0.001 | 0.880 | <0.001 | 0.867 | <0.001 | 0.934 | <0.001 |
| miR-1299 2^-ΔCt^ | 0.721 | <0.001 | 0.678 | <0.001 | 0.690 | <0.001 | 0.796 | <0.001 |
| miR-182-5p 2^-ΔCt^ | 0.968 | <0.001 | 0.961 | <0.001 | 0.949 | <0.001 | 0.980 | <0.001 |
| miR-30e-3p 2^-ΔCt^ | 1.000 |  | 1.000 |  | 1.000 |  | 1.000 |  |
| miR-126-3p 2^-ΔCt^ | 0.973 | <0.001 | 0.970 | <0.001 | 0.958 | <0.001 | 0.981 | <0.001 |
| Waist circumference (cm) | -0.538 | 0.008 | -0.534 | 0.009 | -0.729 | <0.001 | -0.465 | 0.025 |
| Hip circumference (cm) | -0.084 | 0.703 | 0.049 | 0.826 | -0.174 | 0.426 | -0.001 | 0.996 |
| Waist to Hip ratio | -0.083 | 0.705 | -0.035 | 0.874 | -0.018 | 0.934 | -0.144 | 0.513 |
| Systolic blood pressure (mmHg) | 0.275 | 0.204 | -0.017 | 0.940 | 0.342 | 0.110 | 0.408 | 0.053 |
| Diastolic blood pressure (mmHg) | 0.202 | 0.354 | 0.105 | 0.633 | 0.260 | 0.231 | 0.268 | 0.216 |
| Fasting Blood glucose (mmol/L) | 0.119 | 0.590 | 0.205 | 0.347 | 0.257 | 0.236 | 0.006 | 0.978 |
| 2-hour glucose (mmol/L) | 0.058 | 0.792 | 0.211 | 0.335 | 0.263 | 0.226 | -0.124 | 0.572 |
| HbA1c (%) | -0.153 | 0.485 | -0.110 | 0.616 | 0.022 | 0.919 | -0.192 | 0.379 |
| Fasting insulin (mIU/L) | 0.250 | 0.250 | 0.398 | 0.060 | 0.182 | 0.407 | 0.043 | 0.844 |
| 2-hour insulin (mIU/L) | 0.045 | 0.838 | 0.231 | 0.290 | 0.012 | 0.957 | -0.080 | 0.717 |
| Triglycerides-S (mmol/L) | -0.024 | 0.915 | -0.052 | 0.813 | 0.099 | 0.654 | 0.035 | 0.875 |
| Total cholesterol (mmol/L) | 0.015 | 0.945 | 0.036 | 0.871 | 0.165 | 0.451 | 0.233 | 0.284 |
| HDL-cholesterol (mmol/L) | 0.443 | 0.034 | 0.483 | 0.019 | 0.485 | 0.019 | 0.608 | 0.002 |
| LDL-cholesterol (mmol/L) | -0.025 | 0.908 | 0.012 | 0.958 | 0.112 | 0.609 | 0.165 | 0.451 |
| C-Reactive Protein (mg/L) | 0.173 | 0.429 | 0.248 | 0.254 | 0.163 | 0.458 | 0.151 | 0.492 |
| Gamma GT (IU/L) | 0.063 | 0.775 | -0.066 | 0.763 | 0.445 | 0.033 | 0.136 | 0.536 |
| S-Creatinine (µmol/L) | -0.385 | 0.394 | -0.329 | 0.471 | -0.539 | 0.212 | -0.277 | 0.547 |
| Duration of disease (years) | - | - | - | - | - | - | 0.317 | 0.489 |

**Table S5.** Age, gender and BMI adjusted partial correlation coefficients for the association between miR-126-3p relative expression and anthropometric and biochemical parameters according to blood pressure status

|  | **All** | | **Normotensive** | | **Screen-detected HPT** | | **Known HPT** | |
| --- | --- | --- | --- | --- | --- | --- | --- | --- |
|  | r | *p-value* | r | *p-value* | r | *p-value* | r | *p-value* |
| miR-30a-5p 2^-ΔCt^ | 0.901 | <0.001 | 0.891 | <0.001 | 0.878 | <0.001 | 0.915 | <0.001 |
| miR-1299 2^-ΔCt^ | 0.731 | <0.001 | 0.678 | <0.001 | 0.697 | <0.001 | 0.807 | <0.001 |
| miR-182-5p 2^-ΔCt^ | 0.983 | <0.001 | 0.974 | <0.001 | 0.968 | <0.001 | 0.982 | <0.001 |
| miR-30e-3p 2^-ΔCt^ | 0.973 | <0.001 | 0.970 | <0.001 | 0.958 | <0.001 | 0.981 | <0.001 |
| miR-126-3p 2^-ΔCt^ | 1.000 |  | 1.000 |  | 1.000 |  | 1.000 |  |
| Waist circumference (cm) | -0.535 | 0.009 | -0.539 | 0.008 | -0.748 | <0.001 | -0.456 | 0.029 |
| Hip circumference (cm) | -0.053 | 0.809 | 0.104 | 0.636 | -0.134 | 0.542 | 0.041 | 0.852 |
| Waist to Hip ratio | -0.113 | 0.608 | -0.088 | 0.689 | -0.073 | 0.741 | -0.163 | 0.456 |
| Systolic blood pressure (mmHg) | 0.293 | 0.174 | -0.038 | 0.863 | 0.360 | 0.092 | 0.384 | 0.070 |
| Diastolic blood pressure (mmHg) | 0.217 | 0.319 | 0.068 | 0.759 | 0.279 | 0.197 | 0.256 | 0.239 |
| Fasting Blood glucose (mmol/L) | 0.077 | 0.728 | 0.173 | 0.430 | 0.192 | 0.380 | -0.040 | 0.855 |
| 2-hour glucose (mmol/L) | 0.019 | 0.932 | 0.177 | 0.420 | 0.206 | 0.346 | -0.159 | 0.468 |
| HbA1c (%) | -0.170 | 0.439 | -0.094 | 0.670 | -0.006 | 0.977 | -0.230 | 0.292 |
| Fasting insulin (mIU/L) | 0.241 | 0.267 | 0.382 | 0.072 | 0.204 | 0.351 | 0.045 | 0.837 |
| 2-hour insulin (mIU/L) | 0.024 | 0.913 | 0.206 | 0.345 | 0.013 | 0.955 | -0.082 | 0.711 |
| Triglycerides-S (mmol/L) | 0.018 | 0.935 | 0.017 | 0.940 | 0.196 | 0.371 | 0.048 | 0.827 |
| Total cholesterol (mmol/L) | 0.019 | 0.930 | 0.048 | 0.829 | 0.185 | 0.398 | 0.270 | 0.212 |
| HDL-cholesterol (mmol/L) | 0.401 | 0.058 | 0.431 | 0.040 | 0.449 | 0.031 | 0.595 | 0.003 |
| LDL-cholesterol (mmol/L) | -0.017 | 0.939 | 0.031 | 0.889 | 0.137 | 0.534 | 0.209 | 0.339 |
| C-Reactive Protein (mg/L) | 0.139 | 0.528 | 0.184 | 0.401 | 0.174 | 0.426 | 0.122 | 0.580 |
| Gamma GT (IU/L) | 0.047 | 0.830 | -0.080 | 0.717 | 0.440 | 0.035 | 0.116 | 0.598 |
| S-Creatinine (µmol/L) | -0.390 | 0.387 | -0.346 | 0.447 | -0.503 | 0.250 | -0.281 | 0.542 |
| Duration of disease (years) | - | - | - | - | - | - | 0.254 | 0.583 |
